# Supplementary material for: ProteinShader: illustrative rendering of macromolecules
Source: BMC Struct Biol. 2009 Mar 30;9:19. doi: 10.1186/1472-6807-9-19 (PMC2672931; doi:10.1186/1472-6807-9-19)
Supplement: Additional file 1 — ProteinShader program without source code. This compressed file contains the complete ProteinShader program including associated libraries, but no source code. A README.txt file gives an overview of the ProteinShader distribution, and the index.html file in the help subdirectory has directions on getting started with the program as well as a set of tutorials. [file 1472-6807-9-19-S1.zip › ProteinShader-beta-0_9_4-binary/help/api/org/proteinshader/gui/listeners/controlpanel/MotionPanelListenerFactory.html]

MotionPanelListenerFactory (ProteinShader API)


|  |  |  |
| --- | --- | --- |
| **Overview** \| **Package** \| **Class** \| **Use** \| **Tree** \| **Deprecated** \| **Index** \| **Help** | |  |
| **PREV CLASS**   **NEXT CLASS** | **FRAMES**    **NO FRAMES**     **All Classes** |  |
| SUMMARY: NESTED \| FIELD \| CONSTR \| METHOD | DETAIL: FIELD \| CONSTR \| METHOD |  |


---


## org.proteinshader.gui.listeners.controlpanel Class MotionPanelListenerFactory

```
java.lang.Object
  org.proteinshader.gui.listeners.controlpanel.MotionPanelListenerFactory
```

---

``` public class MotionPanelListenerFactory extends Object ```

Creates the listeners for the MotionPanel.

---

**Constructor Summary**

`MotionPanelListenerFactory(Mediator mediator,
MotionPanel motionPanel)`
  
          Constructs a MotionPanelListenerFactory.


| **Method Summary** | |
| --- | --- |
| `ActionListener` | `createStartButtonActionListener()`             Creates an action listener for the 'Start' button. |
| `ActionListener` | `createStopButtonActionListener()`             Creates an action listener for the 'Stop' button. |
| `ActionListener` | `createTextFieldActionListener()`             Creates an action listener for the 'X-Axis' text field. |
| `ChangeListener` | `createXAxisSliderChangeListener()`             Creates a change listener for the 'X-Axis' slider that is used to adjust the rotation speed. |
| `ChangeListener` | `createYAxisSliderChangeListener()`             Creates a change listener for the 'Y-Axis' slider that is used to adjust the rotation speed. |

**Methods inherited from class java.lang.Object**

`clone, equals, finalize, getClass, hashCode, notify, notifyAll, toString, wait, wait, wait`

**Constructor Detail**

### MotionPanelListenerFactory

```
public MotionPanelListenerFactory(Mediator mediator,
                                  MotionPanel motionPanel)
```

:   Constructs a MotionPanelListenerFactory.

    **Parameters:**: `mediator` - the centralized Mediator object that listeners call on to accomplish their task.: `motionPanel` - the MotionPanel that this class creates listeners for.


**Method Detail**

### createStartButtonActionListener

```
public ActionListener createStartButtonActionListener()
```

:   Creates an action listener for the 'Start' button.

    :   **Returns:**: The action listener.

---


### createStopButtonActionListener

```
public ActionListener createStopButtonActionListener()
```

:   Creates an action listener for the 'Stop' button.

    :   **Returns:**: The action listener.

---


### createTextFieldActionListener

```
public ActionListener createTextFieldActionListener()
```

:   Creates an action listener for the 'X-Axis' text field.

    :   **Returns:**: The action listener.

---


### createXAxisSliderChangeListener

```
public ChangeListener createXAxisSliderChangeListener()
```

:   Creates a change listener for the 'X-Axis' slider that is used to
    adjust the rotation speed. The MotionPanel will be told to update
    the 'X-Axis' text field below the slider, and, if an animation is
    in progress, the new speed will also be forwarded to the Mediator.

    :   **Returns:**: The change listener.

---


### createYAxisSliderChangeListener

```
public ChangeListener createYAxisSliderChangeListener()
```

:   Creates a change listener for the 'Y-Axis' slider that is used to
    adjust the rotation speed. The MotionPanel will be told to update
    the 'Y-Axis' text field below the slider, and, if an animation is
    in progress, the new speed will also be forwarded to the Mediator.

    :   **Returns:**: The change listener.


---


|  |  |  |
| --- | --- | --- |
| **Overview** \| **Package** \| **Class** \| **Use** \| **Tree** \| **Deprecated** \| **Index** \| **Help** | |  |
| **PREV CLASS**   **NEXT CLASS** | **FRAMES**    **NO FRAMES**     **All Classes** |  |
| SUMMARY: NESTED \| FIELD \| CONSTR \| METHOD | DETAIL: FIELD \| CONSTR \| METHOD |  |


---

# *Copyright © 2007-2008*
